# Laser Powder Bed Fusion of Chromium Bronze Using Recycled Powder

**DOI:** 10.3390/ma14133644

**Published:** 2021-06-30

**Authors:** Ivan A. Pelevin, Maxim A. Burmistrov, Dmitriy Yu. Ozherelkov, Alexander S. Shinkaryov, Stanislav V. Chernyshikhin, Alexander A. Gromov, Anton Yu. Nalivaiko

**Affiliations:** 1 Catalysis Lab, National University of Science and Technology MISIS, 119991 Moscow, Russia; i.pelevin@misis.ru (I.A.P.); 9851144578@mail.ru (M.A.B.); d.ozherelkov@gmail.com (D.Y.O.); shinkaryov@gmail.com (A.S.S.); a.gromov@misis.ru (A.A.G.); 2 Technical Directorate, AddSol Manufacturing Company, 115201 Moscow, Russia; 3 Center for Design, Manufacturing and Materials, Skolkovo Institute of Science and Technology, 121205 Moscow, Russia; stanislav.chernyshikhin@skoltech.ru

**Keywords:** selective laser melting, laser powder bed fusion, selective laser melting, bronze, chromium bronze, mechanical properties

## Abstract

Laser powder bed fusion (LPBF) of Cu-0.5Cr was carried out using recycled powder taken out from the LPBF machine after previous printing. Various volumetric defects characterized the powder wherein particle size distribution was the same as virgin powder. Using recycled powder resulted in extra spherical pore formation after the LPBF process. Despite that, a relative density of 99.2% was achieved by LPBF parameters optimization. Solidified microstructure with a small volume of defects consisted of an oversaturated dendritic Cu matrix and nano-sized Cr precipitations providing strengthening mechanism occurrence. The possibility of a satisfactory level of mechanical properties with σ_0.2_ = 136.8 MPa, UTS = 187.4 MPa, along with 15.5% of elongation achieving, was shown.

## 1. Introduction

Such ancient material as bronze is still used nowadays in various industries due to its physical and mechanical properties [1]. Excellent electrical and thermal conductivity, corrosion resistance, and high strength and ductility make different bronzes irreplaceable [2]. There are few different alloys based on Cu, which is called bronze. Cu-Sn tin bronze is one of the most commonly used. Sn content varies in a wide range from 0–2 to 15 wt% and even more. Alloys with low (less than 5 wt%) tin content are used in electrics and electronics having relatively high strength [1,3,4]. High-tin bronzes with 10–15 wt% of Sn possess excellent mechanical properties, wear, and corrosion resistance that are useful for marine and bearing applications [5,6]. The addition of a small amount (up to 5 wt%) of chromium to copper leads to significant strength improvement because the common precipitation hardening mechanism is implemented in Cu-Cr alloys [7,8,9,10].

Laser powder bed fusion (LPBF), also known as selective laser melting (SLM), is a novel method of additive manufacturing that consists of melting powder of material by laser beam layer by layer according to the 3D model. To date, a large number of materials are successfully adapted to LPBF, including steels, aluminum, titanium alloys, and many more [11,12,13,14]. Copper and its alloys are of great difficulty to the LPBF process because of high reflectivity, heat conductivity, and poor laser absorption, but it was overcome, and even pure copper was synthesized [15,16,17]. LPBF of Cu-Sn alloys was studied intensively within whole range of tin content: Cu-4Sn [18], Cu-4.3Sn [19], Cu-10Sn [1,20,21], Cu-15Sn alloy [22], Cu-15Ni-8Sn [23]. Casting and mechanical alloying of Cu-Sn alloys provide strengthening also through fabricating in-situ nanostructured grains or nanocomposites [24,25]. The microstructure of LPBFed material is usually even finer compared with cast material because of higher cooling rates 10^2^–10^4^ K/s for LPBF [26] and 10^2^ K/s for mold casting [27]. Rapid solidification providing ultrafine grain structure during the LPBF procedure makes this novel technological approach suitable and promising for implementing high-performing strengthened materials.

In addition to Cu-Sn, Cu-Al-Ni-Mn [28] and Cu-Ni-Si [29] alloys are also in focus, while there is a lack of information about Cu-Cr bronzes known as precipitation hardening copper alloys. Dobatkin et al. [30] used severe plastic deformation to form ultrafine-grained microstructures in Cu–Cr alloys to obtain high material strength and good electrical conductivity. Study of Cu–Cr alloy microstructure, electrical and mechanical properties after LPBF was performed only by Uchida et al. [31]. Specimens exhibited >99.7% density and 640 and 777 MPa tensile strength for the 1.3 Cr, and 2.5 Cr annealed at 450 °C specimens, respectively. As-fabricated tensile strength was 280 and 449 MPa for the 1.3 Cr and 2.5 Cr, respectively. It is also necessary to mention that Ma et al. [32] showed uniformly distributed Zr and Cr precipitations in Cu-Cr-Zr alloy after LPBF providing decreasing distance between them, thus effective strengthening. Popovich et al. [33] studied LPBF of Cu-Cr-Zr-Ti (0.50–0.70 Cr) alloy, and 195–211 MPa ultimate tensile strength and 11–16% elongation were obtained. In addition, much higher plasticity of LPBFed materials than wrought was found [32] due to no post-processing after LPBF resulting in a copper matrix with excellent plasticity.

Additive manufacturing in general and the LPBF method, in particular, are economically beneficial in many cases but costs of initial powder production are still high. Good sphericity, narrow size distribution, and precise chemical composition require complex approaches to their achievement. The LPBF procedure implies less material waste than substractive methods, but reasonable consumption arises when unmelted powder that did not take part in the building is not used in subsequent syntheses. Usage of such recycled powder provides a decrease of the whole LPBF process costs but needs the particular focus of researchers to study this powder applicability. Different defects in the morphology of recycled powder compared with virgin powder that emerged during LPBF can occur [34]. Despite that, a satisfactory level of mechanical properties and density of AlSi10Mg, Inconel 718, Scalmalloy, and Ti6Al4V samples fabricated from the recycled powder was achieved [34,35]. The present research focuses on features of the laser powder bed fusion process of low chromium Cu-Cr alloy (0.5–0.7 wt% of Cr), solidification process, and microstructure formation using recycled powder. In addition, a comparative analysis of the mechanical behavior of the Cu-0.5Cr bronze synthesized using recycled and virgin powder based on experimental data from [36] was performed.

## 2. Materials and Methods

Chromium bronze recycled powder was received from the AddSol Manufacturing Company. Virgin powder was gas atomized into powder with an average particle size of 45 μm. The chemical composition of the powder is presented in Table 1.

The recycled powder was collected after ten printing processes using 5 kg of virgin powder each, 50 kg total. The total amount of recycled powder left after ten printing processes was 45 kg. The amount of powder separated after the sieving with 60 μm mesh size was 0.5 kg. Thus the final amount of recycled powder received for this study was 44.5 kg. The amount of recycled powder used for samples printing investigated in this study was 20 kg (4 printing procedures).

The maximum distance between the printed part and powder layer edge was 50 mm. The LPBF procedure was carried out on an AddSol D250 (AddSol, Moscow, Russia) device equipped with a Yb laser of 400 W power with 1064 nm wavelength and an inverse Gaussian distribution spot of 105 μm diameter. Samples were printed on stainless steel substrates without their preheating under an argon atmosphere. Laser power P = 360 W, layer thickness t = 40 μm, hatch spacing h = 100 μm, and chessboard laser scan strategy with 67° rotation layer by layer were set at varying scanning speeds V = 300–1000 mm/s.

Cubic 10 mm × 10 mm × 10 mm samples were built for structural investigations, while gentel-shaped samples were printed to perform tensile tests.

A Carl Zeiss Axio Observer A1M optical microscope (Carl Zeiss, Jena, Germany) was used for microstructure and porosity analysis. Chemical composition, powder morphology, and microstructure characterization were performed on scanning electron microscopy (SEM) TESCAN Vega 3 (Tescan Analytics, Fuveau, France). Tensile mechanical properties were measured on INSTRON 5569 (Instron, Norwood, MA, USA) using tailored adapters with a 1 mm/min strain speed at room temperature. The digital image correlation analysis (DIC) was performed during a tensile test using a Correlated Solutions VIC-3D testing complex.

## 3. Results and Discussion

**Figure 1:**
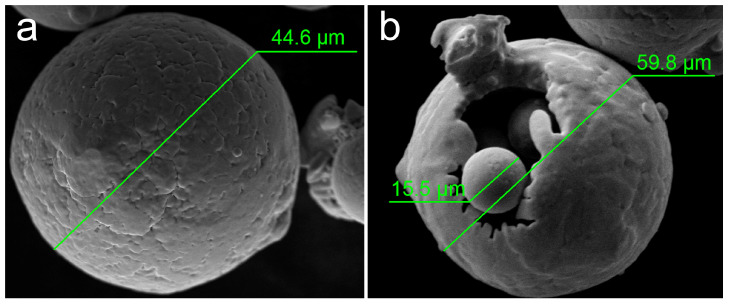
SEM image of typical morphology of virgin (**a**) and recycled (**b**) powder.

Sieving of the raw recycled powder, which did not take part in fusion and was taken from the LPBF machine, allowed to reject large particles. Such large particles arise because of not melting but heating enough to sinter from several small particles. Thus, the size distribution of the recycled powder after sieving is almost the same as with the virgin one. However, some defects related to shape morphology were detected. Large cavities, sintered satellites, and low sphericity of some particles are seen in Figure 1.

**Figure 2:**
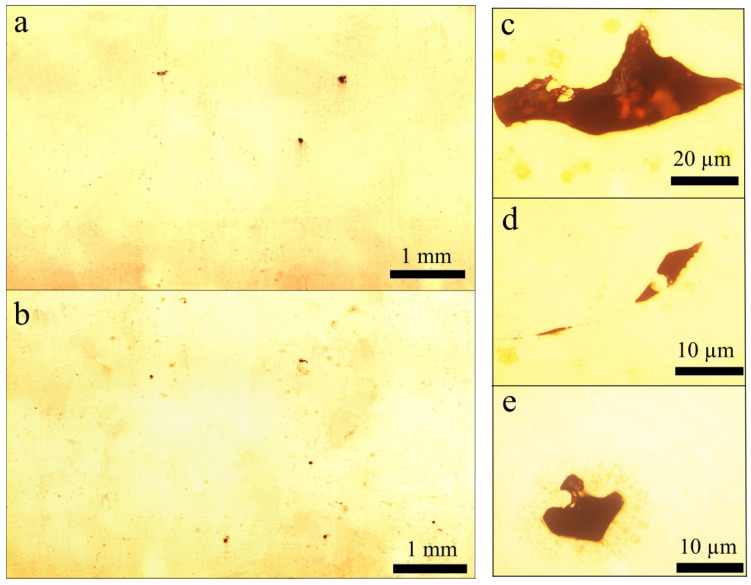
Cross-section of XY (**a**) and XZ (**b**) planes of the most dense sample and typical irregular pores (**c**–**e**).

The cavities with small particles “stuck” inside them are shown in Figure 1. Complicated particle shape leads to decreasing the recycled powder flowability that obstructs proper powder layer formation. Moreover, mentioned defects negatively affect bulk density resulting in uneven filling of the volume with material, thus in irregular heat gradient during the melting process, making it discontinuous. All of that could finally result in the pore formation of solidified material [37] and was present in the microstructure of studied samples. Polished XY and XZ planes of the built cubic sample and typical pores are presented in Figure 2.

The regime of LPBF, i.e., energy density (ED), was varied by changing the laser scanning speed to find regularities between density, mechanical properties, and ED, thus revealing optimal synthesis conditions providing the highest possible properties. Energy density is a convenient parameter for evaluation and comparison of the LPBF regimes, which represents energy supply to a unit of volume of the material and is expressed as:

(1) $$\mathrm{ED}=\frac{P}{\mathrm{Vht}}$$

The described approach allowed to obtain high dense samples with rare pores (see Figure 2a,b) using a recycled powder, which means proper LPBF regime selection. The porosity of the sample LPBFed with scanning speed V = 500 mm/s was found to be 0.8% while decreasing (up to 300 mm/s) and increasing (up to 1000 mm/s) both affect porosity negatively, making sample density far below 99%.

The process of LPBF is combined from two main aspects: the wetting behavior of metal and the coalescence of melted powder particles. During the process, the input laser energy must be sufficient to melt both particles and the substrate. Then, melted particles are pulled towards the substrate, driven by the surface tension. As a result, forming a continuous and defect-free metal layer requires the uniform spread of molten metal across the substrate surface. The described wetting effect is crucial for the 3D printing of material with high mechanical properties. The sample cross-section contains pores of various sizes (see Figure 2c–e) but with similar geometry. Such irregularly shaped pores result from poor wetting behavior. The main factor of such pores formation is the surface roughness of the previously solidified layer affecting the next powder layer’s thickness. The defects of the recycled powder determine such roughness shown in Figure 1b. During the printing process, the pores induced by the previous layer’s roughness will not be filled with the liquid phase, inducing the formation of irregular pores after the solidification of the melt pool.

**Figure 3:**
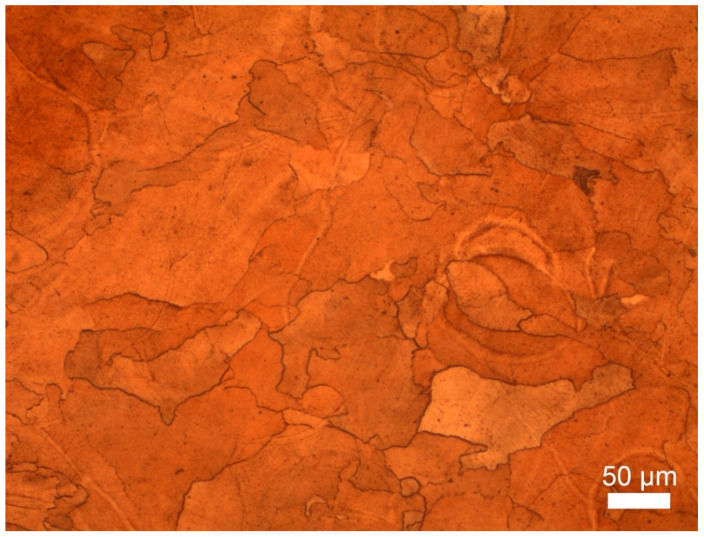
Etched microstructure of the most dense sample fabricated by the optimal regime.

The grain structure, which is clearly seen from Figure 3, is similar to that in [31], but grains in the present study are remarkably coarser. It could be explained by lower chromium content, thus fewer precipitations content in the structure and in turn fewer nucleation centers and impediments to grain growth. As long as the chromium melting point (1857 °C) is far higher than the copper one (1083 °C), Cr solidification and precipitation occur first, and then Cu grains form during the cooling process. Moreover, lower chromium precipitation quantity means fewer barriers for copper grains growth. The grain structure of the as-print sample is non-equiaxed, which is typical for Cu-Cr alloys [31].

**Figure 4:**
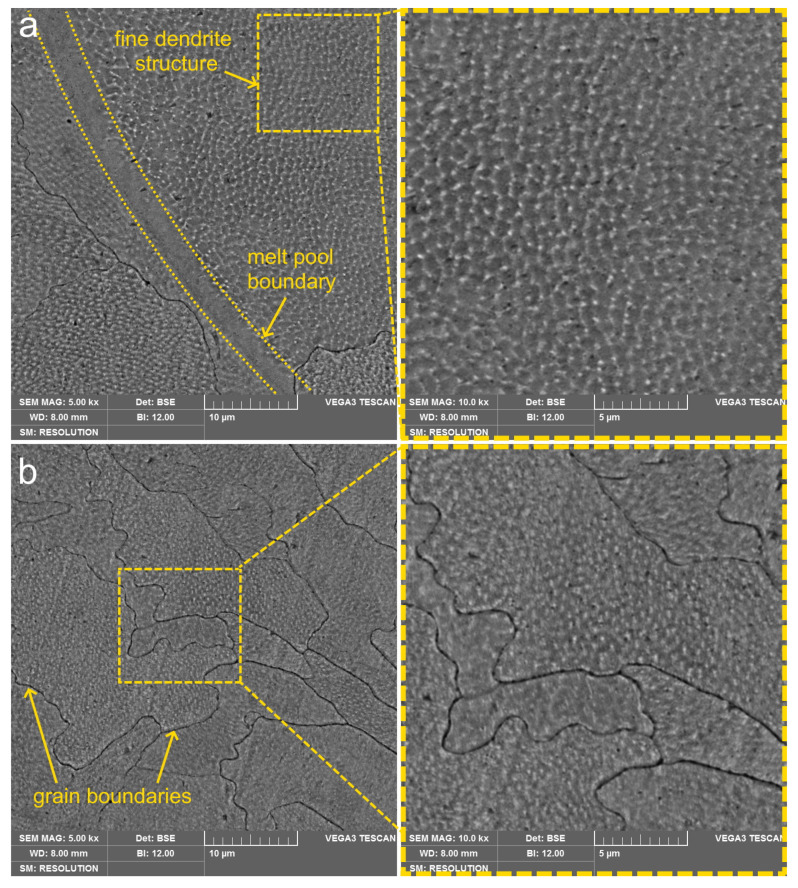
Fine dendritic structure after LPBF with clearly seen melt pool (**a**) and grain (**b**) boundaries.

By the results of SEM analysis in the Z plane with higher magnification (see Figure 4), the dendritic patterns were observed. The obtained microstructure exhibited clear grain boundaries, scan track paths, and melt pool boundaries typical for the SLM process. The dendritic and grain growth was overlapped across several scan layers, which indicated the epitaxial growth character of grains and dendrites through the scan layers. The fine structure with dendrite size less than 1 µm indicated very high solidification rates that have occurred during the SLM process. The grain pattern of the material is mostly equiaxed and inequigranular with 15–150 µm grain size.

Tensile properties of all synthesized samples using recycled powder were examined, and the results are listed in Table 2. Both yield strength (σ_0.2_) and ultimate tensile strength (UTS) are expectedly slightly lower than obtained by Grigoryants et al. [36] using virgin atomized powder at the same LPBF machine and synthesis regimes, while the elongation of the best sample in the present study is even higher.

**Figure 5:**
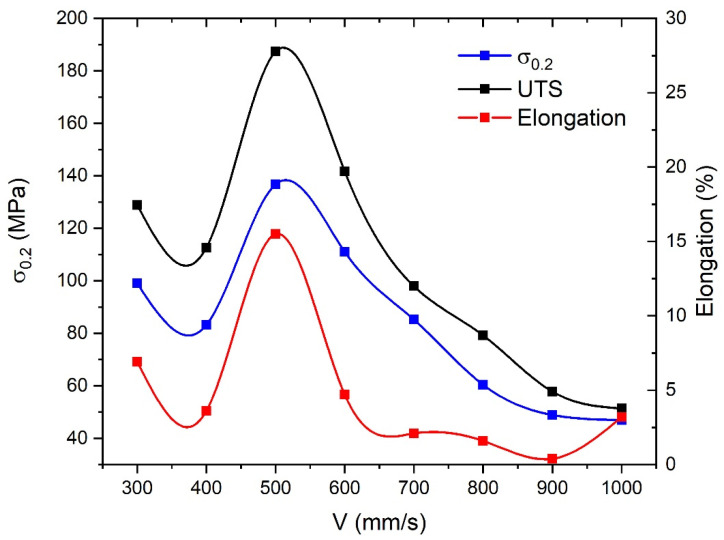
Dependences of ultimate tensile strength and yield strength on scanning speed.

Peaks of elongation, yield and ultimate strength observed in Figure 5 correlate with the dependence of porosity on scanning speed. A higher volume of pores facilitates crack formation and propagation, thus accelerating the fracture of the material. Both grain refinement and precipitation strengthening by chromium addition into the Cu matrix provide high strength and superior ductility and elongation due to the matrix [8,10]. Extremely high cooling rates inherent in LPBF provide ultra-fine grain structure and, thus, grain-boundary strengthening according to with Hall–Petch relation, wherein solid solution strengthening, according to Liu et al. [38], should not be expected. Cr is distinguished by low solubility in copper, which is beneficial to the strengthening effect. Rapid solidification of the melt results in super-saturated solid solution and some volume of Cr precipitates as nano-sized particles providing proper strengthening of as-built samples. Furthermore, the high wettability of chromium with the melt and solid copper afforded uniform distribution of precipitated particles within melt and solidified structure and well interface bonding. Uniform distribution of precipitates is of great importance as agglomerations and segregations increase distances between particulates, i.e., dislocation movement barriers, decreasing resultant mechanical properties.

**Figure 6:**
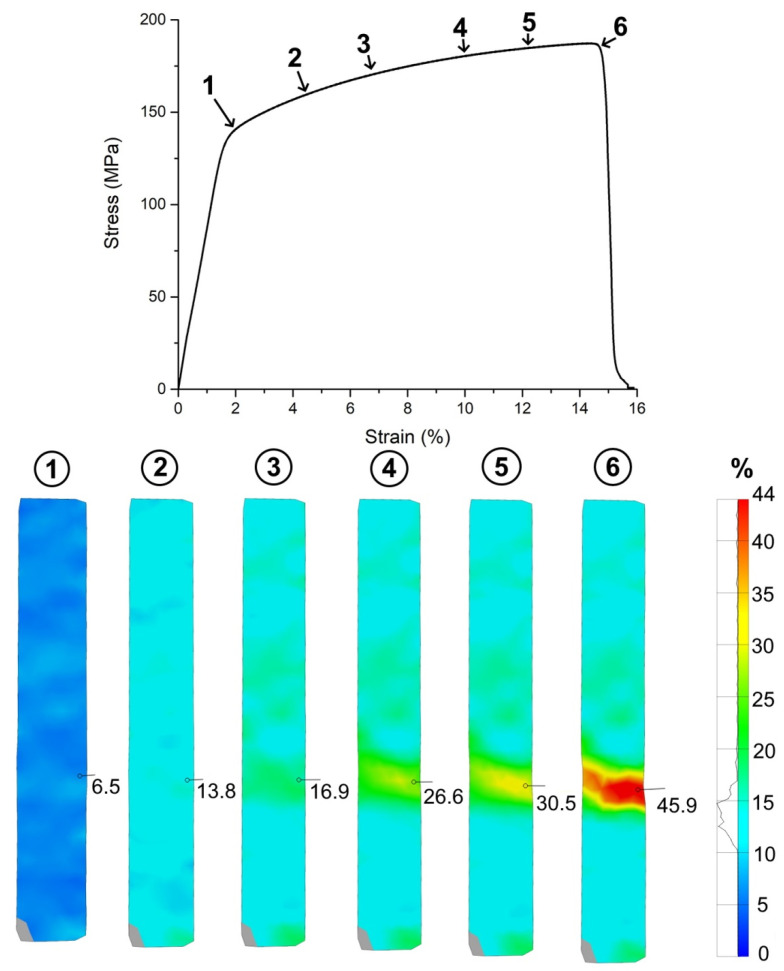
Typical stress–strain curve combined with DIC analysis; 1–6: DIC analysis at corresponding points on the stress-strain curve.

The typical stress–strain curve for the samples obtained with optimized SLM parameters (V = 500 mm/s) is presented in Figure 6. To illustrate the material’s tensile properties, the analysis of deformation behavior by the DIC method was presented and combined with the stress–strain curve.

**Figure 7:**
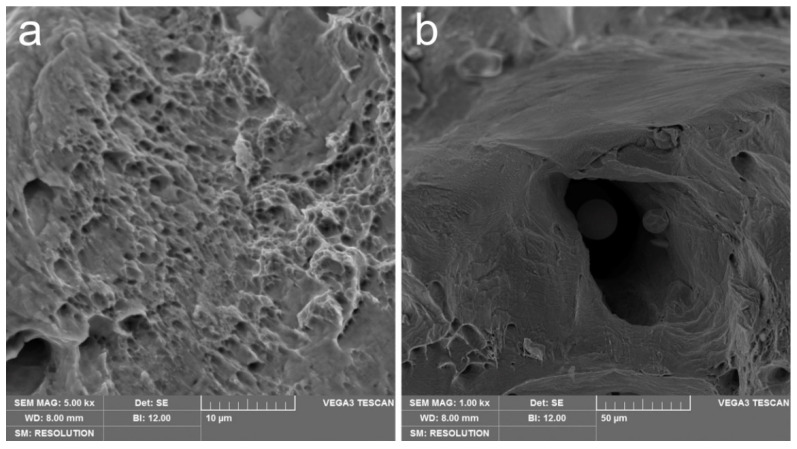
SEM of Cu-0.5Cr bronze after the tensile test: typical dimple structure (**a**) and pore with unmelted particles inside (**b**).

The tensile behavior is characterized by a typical ductile fracture of the sample with high elongation numbers (up to 15%) and contraction. According to DIC analysis, the local deformations before fracture were around 45% and were localized in the fracture area of 5 mm. The SEM analysis of the fracture surface confirmed ductile fracture behavior (see Figure 7).

Dimple structure inherent in the ductile fracture is discerned in Figure 7a. The size of the dimples is mainly less than the grain size, which indicates intergrain fraction spread. The mechanism responsible for the small voids initiation is based on fine dendritic structure and movement of dislocations arising from dendritic boundaries. Pores also participate in fracture, facilitating the process. SEM confirms pore formation because of recycled powder usage (see Figure 7b). Unmelted spherical particles inside the pore are detected and have a similar size with satellites observed for recycled powder (see Figure 1b).

**Figure 8:**
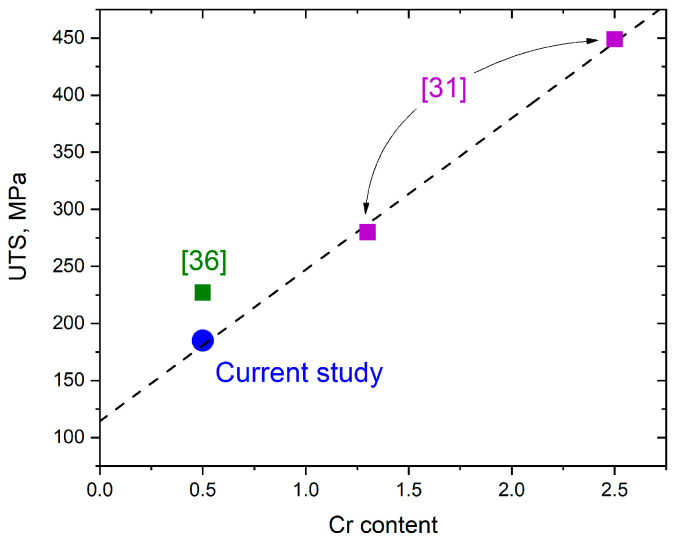
Comparison of UTS of Cu-Cr alloys depends on the chromium content obtained by Uchida et al. [31], Grigoryants et al. [36], and the current study.

They recycled powder usage for the LPBF process of Cu-0.5Cr bronze resulted in strength reduced by approx. 17% compared with virgin powder usage [36] (see Figure 8), but still providing a satisfactory UTS level. The highest UTS of the as-built sample using virgin powder was 227 MPa, while recycled powder provides UTS = 187.4 MPa. As LPBF regimes were the same for both powders, such strength reduction is mainly associated with extra pore formation because of defects in the recycled powder mentioned above (see Figure 1). The presence of the pores critically affects crack formation, and propagation thus decreasing the final mechanical properties.

Considering the low chromium content of the studied alloy and as-fabricated UTS using virgin powder from [31], linear dependence on Cr content appears (see Figure 8). The precipitation strengthening mechanism is highly affected by precipitation phase/element content, resulting in different particles in the structure, so the linear dependence observed in Figure 8 is expected. It makes the obtained UTS of 187.4 MPa using recycled powder a satisfactory level, especially when combined with superior elongation of 15.5%, which is significantly higher than the results of previous works [31].

## 4. Conclusions

Laser powder bed fusion of chromium bronze with 0.5 mass.% was successfully performed using a recycled powder that was not used in the previous LPBF process, taken from the machine, and sieved to reject large particles. Defects of particle shape characterized the recycled powder, i.e., cavities, sintered satellites, low sphericity of some particles were observed. The particle size distribution of recycled powder after sieving was the same as virgin powder. The defects in powder led to extra pore formation in solidified material. Despite that, a near full dense sample with RD = 99.2% was obtained using the optimal LPBF process regime: V = 500 mm/s, P = 360 W, layer thickness of 40 μm, hatch spacing of 100 μm and chessboard laser scan strategy with 67° rotation layer by layer. The formation of an almost defect-free microstructure consisted of fine copper dendritic matrix oversaturated by chromium with nano-sized chromium precipitations due to rapid solidification during the LPBF process. Pore formation was induced by the previous layer’s roughness that was not filled with the liquid metal. Thus, the precipitation strengthening mechanism was observed, providing a satisfactory level of mechanical properties: σ_0.2_ = 136.8 ± 8.7 MPa, UTS = 187.4 ± 10.1 MPa, along with 15.5 ± 2.3% of elongation, which is in good agreement with the literature data of the similar alloys with higher chromium content. According to DIC analysis, the ductile fracture was observed with local deformations up to 45%, despite using recycled powder with defects in morphology. Thus, it demonstrates the fundamental possibility of recycled powder usage in Cu-Cr bronze synthesis by the LPBF approach.

## Figures and Tables

**Table 1:** Chemical composition of the initial powder, mass %.

Cu	Cr	Fe	Zn	Impurities
Bal.	0.5	<0.08	<0.3	<0.5

**Table 2:** Mechanical properties of Cu-0.5Cr alloy fabricated using recycled powder depending on laser scanning speed.

V, mm/s	σ_0.2_, MPa	UTS, MPa	Elongation, %
300	99.0 ± 6.3	125.7 ± 9.2	6.9 ± 1.2
400	83.2 ± 11.1	112.6 ± 7.5	3.62 ± 0.8
500	136.8 ± 8.7	187.4 ± 10.1	15.5 ± 2.3
600	111.0 ± 10.9	141.7 ± 8.0	4.7 ± 1.1
700	85.2 ± 7.0	97.9 ± 6.3	2.1 ± 0.9
800	60.4 ± 13.2	79.2 ± 7.8	1.6 ± 0.6
900	48.9 ± 12.6	57.8 ± 11.4	0.4 ± 0.1
1000	47.0 ± 8.8	51.4 ± 12.7	3.2 ± 0.5

## Data Availability

Data sharing is not applicable to this article.
